# Logical Analysis of Data (LAD) model for the early diagnosis of acute ischemic stroke

**DOI:** 10.1186/1472-6947-8-30

**Published:** 2008-07-10

**Authors:** Anupama Reddy, Honghui Wang, Hua Yu, Tiberius O Bonates, Vimla Gulabani, Joseph Azok, Gerard Hoehn, Peter L Hammer, Alison E Baird, King C Li

**Affiliations:** 1 Rutgers Center for Operations Research, RUTCOR, 640 Bartholomew Road, Piscataway, NJ 08854, USA; 2 Molecular Imaging Laboratory, Clinical Center, National Institutes of Health, Bethesda, MD 20892, USA; 3 National Institute of Neurological Disorders and Stroke, Bethesda, MD 20892, USA; 4 Critical Care Medicine Department, Clinical Center, National Institutes of Health, Bethesda, MD 20892, USA; 5 Department of Radiology, The Methodist Hospital, 6565 Fannin Street, Houston, TX 77030, USA

## Abstract

**Background:**

Strokes are a leading cause of morbidity and the first cause of adult disability in the United States. Currently, no biomarkers are being used clinically to diagnose acute ischemic stroke. A diagnostic test using a blood sample from a patient would potentially be beneficial in treating the disease.

**Results:**

A classification approach is described for differentiating between proteomic samples of stroke patients and controls, and a second novel predictive model is developed for predicting the severity of stroke as measured by the National Institutes of Health Stroke Scale (NIHSS). The models were constructed by applying the Logical Analysis of Data (LAD) methodology to the mass peak profiles of 48 stroke patients and 32 controls. The classification model was shown to have an accuracy of 75% when tested on an independent validation set of 35 stroke patients and 25 controls, while the predictive model exhibited superior performance when compared to alternative algorithms. In spite of their high accuracy, both models are extremely simple and were developed using a common set consisting of only 3 peaks.

**Conclusion:**

We have successfully identified 3 biomarkers that can detect ischemic stroke with an accuracy of 75%. The performance of the classification model on the validation set and on cross-validation does not deteriorate significantly when compared to that on the training set, indicating the robustness of the model. As in the case of the LAD classification model, the results of the predictive model validate the function constructed on our support-set for approximating the severity scores of stroke patients. The correlation and root mean absolute error of the LAD predictive model are consistently superior to those of the other algorithms used (Support vector machines, C4.5 decision trees, Logistic regression and Multilayer perceptron).

## Background

Strokes are the third leading cause of morbidity in the United States, affecting nearly 700,000 Americans annually. Two major types of stroke, ischemic stroke (due to blood clotting) and hemorrhagic stroke (due to bleeding inside the brain), require opposite treatments. An early and rapid diagnosis of strokes is critical for proper treatment of the patients. CT scan and MRI are the effective imaging modalities for diagnosis of strokes; however, such expensive tools are available only in specialized hospitals. The objective of this study is to discover biomarkers of ischemic stroke which could potentially be used for diagnosis of ischemic stroke. Once the potential biomarkers are discovered, their amino-sequences can be identified and be used to develop clinical assays in the next phase of the study. Current study is focused on biomarker discovery on ischemic stroke without any comparison with hemorrhagic stroke.

Mass spectrometry is emerging as a key tool for high throughput characterization of peak profiles for clinical serum or plasma samples. The combination of mass spectrometry and bioinformatics has been proven to be an effective means for the discovery of potential multiple biomarkers for various cancers, such as ovarian [1,2], breast [3], and prostate [4] cancers, and other diseases from patient's serum or plasma samples [5,6]. SELDI (Surface-Enhanced Laser Desorption/Ionization) is widely used to acquire the peak profiles from 2 kDa to 200 kDa for a large number of clinical samples. Other high resolution mass spectrometer, such as prOTOF 2000 (PerkinElmer, MA), also has shown promise in the discovery of potential biomarkers for Alzheimer's disease [6]. In this study, we used high throughput SELDI techniques to screen 48 stroke patients and 32 healthy controls in order to discover novel biomarkers for the diagnosis of ischemic stroke. The samples were fractionated with Ciphergen's serum fractionation kit with anion exchange resin in 96-well format into 6 fractions. The peak profiles for 5 fractionated samples were acquired with Ciphergen ProteinChip Reader PBSIIc using IMAC and CM10 biochips. The mass spectra were analyzed with CiphergenExpress3.0 to generate peak maps. Then the Logical Analysis of Data (LAD) methodology was applied to build a classification model for distinguishing the stroke patients from controls. To validate the model, a validation dataset of SELDI data were acquired in the same way as the training dataset from an independent set of 35 stroke patients and 25 healthy controls.

LAD is a data mining method based on combinatorics, Boolean functions, and optimization [7-9] that has been successfully applied to data analysis problems in different domains, including biology and medicine [10-14]. The novelty of the approach presented in this paper is that we use robust pre-processing steps and the LAD methodology to mine proteomic data to discover a minimum sized support-set. A remarkable feature of LAD is its ability to discover not only potential biomarkers but also potential combinatorial biomarkers.

In this study, we restrict our analyses to ischemic stroke. From here on we refer to an ischemic stroke patient simply as a stroke patient.

## Methods and Results

The main aim of this study is to identify a small set of peaks to develop LAD models for the purpose of (i) classifying an individual observation as a stroke patient or as a control, and (ii) predicting the severity score of a stroke patient. In this section, firstly we describe the data. Next, we discuss the pre-processing steps that we applied on this proteomic dataset. Then we introduce the main concepts of Logical Analysis of Data and illustrate them in the context of our analysis of the SELDI data consisting of peak profiles of 48 stroke patients (referred to as "positive" observations) and 32 controls (referred to as "negative" observations). Each stroke patient or control is described as a vector of numerical components, with each value corresponding to a peak in the SELDI MS (mass spectrum). In what follows, we refer to stroke patients and controls simply as observations, and we use the terms peak and variable interchangeably.

### 2.1. Description of the samples and SELDI MS data

Two sets of plasma samples were collected from stroke patients and healthy adult volunteers according to the IRB-approved (Institutional Review Board) protocol. The blood samples of stroke patients were collected in a local hospital (Suburban Hospital, Bethesda, USA). Follow-up samples in 3 and 6 months after stroke were also collected for future studies. Those stroke patients with cardiovascular instability, severe anaemia, hemorrhagic diathesis, current infection or current severe allergic disorders were excluded from the study. Blood samples for healthy control patients were collected in the NIH blood bank (NIH, Bethesda, USA). Healthy volunteers with active medical problems (allergies, allergic disorders or current symptomatic infection) were also excluded from the study. Table 1 shows the distribution of the clinical data (age, gender and sampling time). The values in this table are median ± standard deviation if the variable is numerical, and proportion if the variable is binary.

**Table 1:** The distribution of clinical data

	Training		Validation	
		
	Stroke patients	Controls	Stroke patients	Controls
Number of subject	48	32	35	25
Age	78 ± 13.59	76 ± 7.71	74.5 ± 14.00	75 ± 7.29
Gender (male)	52%	34%	45%	44%
Sampling in 48 hr*	65%	N/A	100%	N/A

* The blood sample was collected after the stroke

The samples were fractionated into 6 fractions with Ciphergen's serum fractionation kit and SELDI mass spectra were acquired for fractions 1, 3, 4, 5 and 6 with Ciphergen's ProteinChip PBSIIc Reader using CM10 and IMAC chips according to the protocols provided by the manufacturer. Same fractionated samples were applied to CM10 and IMAC chips. Each sample was run in duplicate. Each ProteinChip was acquired with two laser settings. Quality controls with 12 pooled samples that were run together with clinical samples indicated that the %CV (Percent coefficient of variation) of the reproducibility was within 20–30% for major 20–30 peaks.

### 2.2. Pre-processing

The SELDI MS data collected for this study contained many peaks, each of which potentially corresponds to the intensity level of a specific protein. In fact, many of these peaks are irrelevant for the recognition of a stroke patient (as opposed to a control). In this subsection, we describe the procedure used for retaining only those peaks which display good properties in terms of shape, reproducibility, and relevance to the problem of distinguishing between stroke patients and controls.

Out of the peaks originally identified by the SELDI mass spectrometer, we initially selected a subset using the peak detection algorithm in CiphergenExpress 3.0. The criteria for selecting peaks are as follows:

Low M/Z focus: S/N = 2.5, valley = 2.0, 10% spectra as 1^st ^pass

          S/N = 1.5, valley = 1.5, 0.3% mass window as 2^nd ^pass

High M/Z focus: S/N = 2.5, valley = 2.5, 10% spectra as 1^st ^pass

          S/N = 2.0, valley = 2.0, 0.3% mass window as 2^nd ^pass

The choice of peak detection thresholds is based on operator's experience to balance the peak number and signal/noise. The peak detection thresholds were set for training set and kept same for validation set. Using the above criteria for selecting peaks, a total of 1,495 SELDI MS peaks were retained. All retained SELDI MS peak intensities were normalized. The standard normalization formula for the value *x*_*ij *_of a variable *x*_*i *_in an observation *j *is *x*_*ij*_*= m*_*i *_+ *σ*_*i *_${x^{\prime }}_{ij}$, where *m*_*i *_is the mean of *x*_*i*_, and *σ*_*i *_is its standard deviation, and ${x^{\prime }}_{ij}$ is the normalized value. In the course of the LAD analysis, we observed that some peaks selected by the peak detection algorithm were either very noisy or poorly defined. The inclusion of such peaks in the LAD model could degrade its significance, as rules derived from noisy data cannot be considered reliable.

This set of 1,495 peaks was further simplified to retain a subset of peaks based on the following filters:

1. Reproducibility: The SELDI technique used to screen 48 stroke patients and 32 healthy controls was repeated twice using two different bioprocessors. Duplication error – which is defined as the proportion of deviation of the measurement from the average – is calculated for each observed value. The peaks which have an absolute duplication error exceeding 30% in more than 30% of the observations are considered to have low reproducibility and are eliminated;

2. Predictive power: High quality combinatorial patterns with characteristics: degree 2, homogeneity of at least 80% and prevalence of at least 80% (see section 2.4 for definition of pattern characteristics) were generated and the peaks participating in these were retained;

3. Quality of spectrum: Each of the peaks that were retained was manually examined in terms of peak shape (whether peaks are significantly pronounced and distinct from each other).

**Figure 1:**
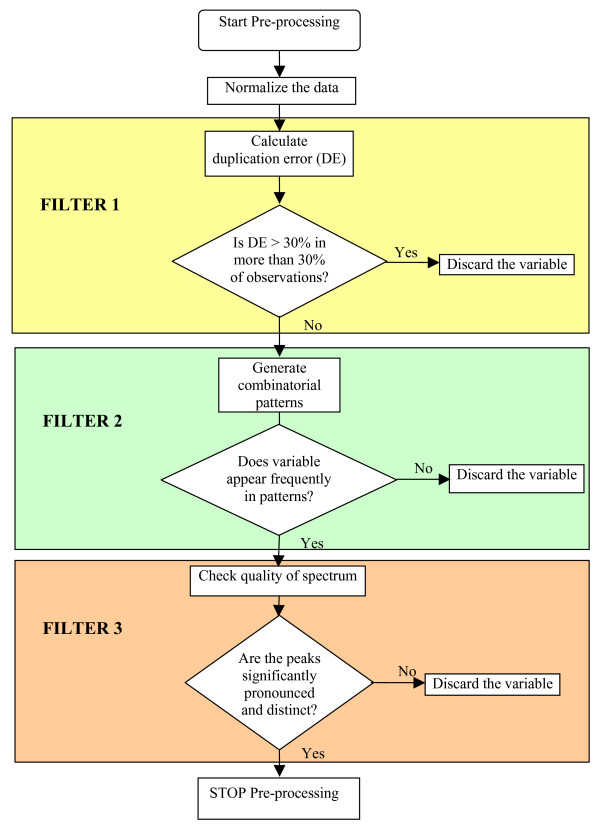
Flowchart for the pre-processing procedure.

After the application of this filtering procedure, we obtained a subset of 96 well-defined, relevant peaks. The flow chart in Figure 1 depicts the above pre-processing steps.

### 2.3. Discretization and Feature Selection

A standard step in the analysis of data containing numerical variables with LAD is the procedure of discretization, in which each numerical variable is assigned a (usually small) number of cut-points, or threshold levels, that represent activity/intensity levels of that variable. A simple interpretation of such a cut-point is that of establishing a threshold value under which a variable is considered to be "low" and above which the variable is considered to be "high." As we shall see later, cut-points are the basis for the synthesis of general rules that can be used for classification and prediction purposes.

For each peak, we have determined one or more relevant cut-points. Virtually any intensity value of a peak can be considered a cut-point. However, we focus on identifying cut-points with a high distinguishing power. For example, -0.15 is such a critical value for peak C043564_: the set of observations for which C043564_ takes a value larger than -0.15 includes 28 of 32 controls, while the set of observations for which C043564_ takes a value less than -0.15 includes 37 out of 48 patients.

We shall associate to a peak *p *and cut-point *c *an "indicator variable" *I(p > c) *defined to take the value 1 on those observations for which the intensity of *p *is greater than *c*, and to take the value 0 on those observations for which the intensity of *p *is less than or equal to *c*. For example, if *p *is the peak C043564_ and -0.15 is a cut-point of *p*, then the corresponding indicator variable *I(C043564_ > -0.15) *is equal to 1 for the observations for which C043564_ exceeds -0.15, and 0 for the other observations.

A feature selection procedure is applied during the discretization process and only a small subset of indicator variables are retained by solving a set-covering model [8]. The set of peaks corresponding to the selected indicator variables is called a "support-set." We selected 5 indicator variables, corresponding to a support-set of 3 peaks, presented in Table 2.

**Table 2:** Peptides in support-set and their corresponding source

Training data		Validation data	
	
Peak ID (M/Z)	Source	Peak ID (M/Z)	Source
C08689_4 (8689 Da)	CM10 chip, Fraction 4, Low noalign Ce3	C08706_6 (8706 Da)	CM10 chip, Fraction 4, Low noalign Ce3
C043564_ (43564 Da)	IMAC chip, Fraction 6, High noalign Ce3	C043560_ (43560 Da)	IMAC chip, Fraction 6, High noalign Ce3
C044761_ (44761 Da)	IMAC chip, Fraction 6, High noalign Ce3	C044684_ (44684 Da)	IMAC chip, Fraction 6, High noalign Ce3

The three peaks used in this model are C08689_4, C043564_, and C044761_. Because of the differences observed in the M/Z values of the peaks measured in the training and validation sets, we show in Table 2 the support-set used in the training set, along with the corresponding matching peaks used in the validation set. Note that the M/Z values obtained in the validation set are not the same as those in the training set, but are still quite close. The M/Z value and the shape of the peak were used as criteria to match them. Indeed, C043560_ in Table 2 (observed in IMAC chip in fraction 6) is the same peak as C043564_ (observed in IMAC chip in fraction 6).

**Figure 2:**
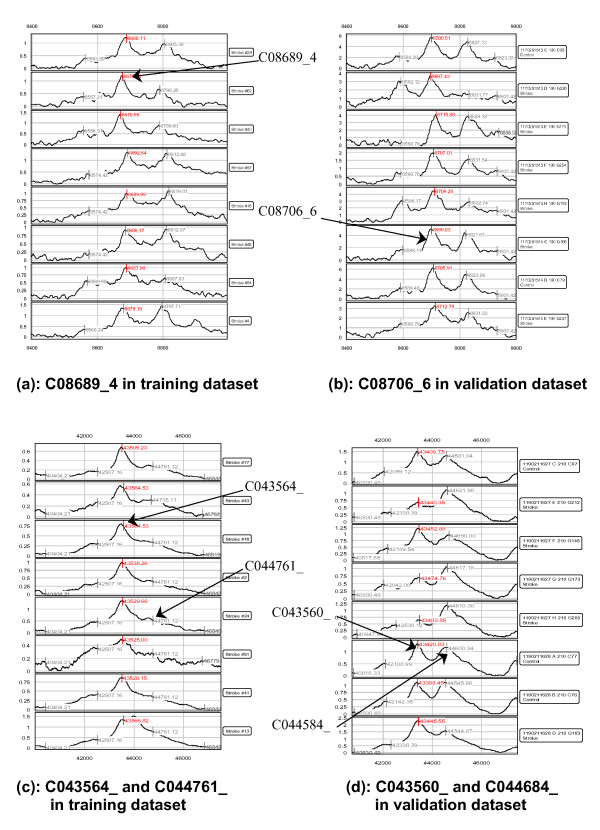
**SELDI peaks for the three potential biomarkers are shown in training and validation sets.** 8 spectra from each dataset were selected randomly to show that peaks in the training set are indeed the same peaks observed in validation set, even though the M/Z value is not identical in both datasets (possibly due to calibration error).

The SELDI MS for these three peaks are shown in Figure 2. Figures 2a and 2b show the typical peak of C08689_4 in the training and validation datasets. Figures 2c and 2d show the typical peaks of C043564_ and C044761_ in the training and validation datasets.

In some datasets, indicator variables alone can separate positive observations from the negative ones. This is not the case in the dataset considered in this study, and for this reason, we consider the use of combinations of indicator variables, as described in the next subsection.

### 2.4. Combinatorial patterns

Below we show how one or more indicator variables can be used in order to produce rules that can define subgroups of interest within the data. While an indicator variable can partially predict the outcome by relating the high or low value of a variable with a specific outcome, the simultaneous use of more than one indicator variable allows for the definition of more complex rules that can be used for the precise classification of an observation. Such rules are called "combinatorial patterns" and can be regarded to be indicative of a specific condition.

Combinations of indicator variables can define homogeneous subgroups of observations with distinctive characteristics. For example, the set of observations which satisfy both "*I(C017328_ > -0.64) *= 0 and *I(C029061_ > 4.63) *= 0" includes 21 (out of the 48) stroke patients and none of the controls. Clearly, this subgroup has a distribution of positive and negative cases which is significantly different than that of the original population. Due to the fact that this set includes a large proportion of stroke patients, and a very low proportion (in this case 0%) of the controls, we shall say the requirement of "*I(C017328_ > -0.64) *= 0 and *I(C029061_ > 4.63) *= 0" defines a "positive combinatorial pattern." For simplicity, we will refer to this simply as a positive pattern. A positive pattern can be interpreted as a cluster of stroke patients defined by some limitation imposed on the intensity of a few (in this case, two) peaks. Moreover, if a new observation (i.e. one not appearing in the given dataset) satisfies the limitations imposed by the definition of this positive pattern, we shall conclude that in all likelihood this observation is a stroke patient. In what follows, if an observation satisfies the definition of a pattern, we shall say that the observation is covered by that pattern.

Similarly, let us consider now the set of observations satisfying the limiting conditions "*I(C06858_3 > 2.44) *= 0 and *I(C014069_ > 0.97) *= 1." This set includes 11 (out of 32) controls and 1 (out of 48) stroke patient. We shall say that these two conditions define a "negative combinatorial pattern," or simply a negative pattern.

We define below six important characteristics of a positive or negative pattern:

1. Degree, defined as the number of variables involved in the definition of the pattern;

2. Positive prevalence, defined as the proportion of positive observations covered by the pattern;

3. Negative prevalence, defined as the proportion of negative observations covered by the pattern;

4. Positive homogeneity, defined as the proportion of positive observations among all those observations covered by the pattern;

5. Negative homogeneity, defined as the proportion of negative observations among all those observations covered the pattern;

6. Hazard ratio, defined as the ratio between the proportion of positive observations among all those observations covered by the pattern, and the proportion of positive observations among those observations not covered by the pattern.

The positive pattern described above (*I(C017328_ > -0.64) *= 0 and *I(C029061_ > 4.63) *= 0) is defined by 2 peptides and thus it is a degree 2 pattern. The set of observations covered by this pattern includes 21 stroke patients and 0 controls. Thus, the positive prevalence of this pattern is 43.75% (21 out of 48 stroke patients), and the negative prevalence is 0% (0 out of 32 controls). The positive homogeneity of this positive pattern is 100%, since it covers only positive observations and its negative homogeneity is 0%. The hazard ratio of this pattern is (21/21)/(27/59) = 2.19.

### 2.5. Classification model

We constructed an *LAD model *using the variables selected in the support-set, with the purpose of classifying new observations. An LAD model is simply a collection of positive and negative patterns of good characteristics, with the property that every observation in the dataset is covered by at least one of the patterns. Ideally, the positive patterns of a model would cover exclusively positive observations, while its negative patterns would cover only negative observations. In real-life applications, however, one can rarely expect to be able to construct such a model. We use a set-covering model (standard combinatorial model) to select the patterns (rules) in the classification model.

The LAD model in Table 3 consists of 3 positive and 2 negative patterns built using the intensity levels of the 3 peptides in the support-set shown in Table 2. It can be seen that:

• Each pattern has at most degree two (i.e. its definition involves at most two peaks);

• In average each positive pattern covers 72% of the patients, and each negative pattern covers 50% of the controls; clearly, each pattern defines a reasonably large subset of patients or controls.

**Table 3:** LAD classification model

Pattern	Degree	Positive Prevalence	Negative Prevalence	Positive Homogeneity	Negative Homogeneity	Hazard Ratio	C08689_4	C043564_	C044761_
P1	1	37 (78.72%)	3 (9.68%)	92.50%	7.50%	3.52		≤ -0.154	
P2	2	35 (74.47%)	4 (12.90%)	89.74%	10.26%	2.92	≤ 0.162		≤ 0.553
P3	1	31 (65.96%)	2 (6.45%)	93.94%	6.06%	2.64	≤ -0.237		
N1	2	3 (6.38%)	20 (64.52%)	13.04%	86.96%	0.16	> 0.162	> -0.154	
N2	1	2 (4.26%)	11 (35.48%)	15.38%	84.62%	0.22			> 0.728

### 2.6. Data cleaning

During the LAD data analysis, two observations (Stroke#61 and Stroke#23) were identified as potential outliers in the dataset. We consider a patient (control) to be an outlier if its measurements are very similar to those of observations in the set of controls (patients). The reason for such an assumption was that both observations were consistently misclassified in preliminary cross-validation experiments. In the presence of the outliers the performance of the LAD classification model drops by approximately 2% in all three measures (accuracy, sensitivity and specificity).

The discovery of these two outliers is a by-product of the cross-validation evaluation of our LAD models. This "by-product" feature is not necessarily inherent to the LAD methodology, but was identified during our LAD experiments. The two samples that were consistently misclassified by our LAD classifiers turned out to be abnormal samples even though the cross-validation LAD experiments were carried out without any prior suspicions.

Upon consulting the original database we confirmed that Stroke#61, a stroke patient in the dataset, was in fact an intra-cerebral haemorrhage (ICH) patient as opposed to an ischemic stroke patient. This sample was mistakenly added to the sample set by one of our collaborators unintentionally. It was discovered through this data cleaning process without prior knowledge. Stroke#23, appearing as a control in the dataset, was in fact a 63-year old Caucasian female, with stroke risk factors of hypertension, prior smoking, prior hormone replacement therapy and hypercholesterolemia. She also has a history of migraine, depression and asthma as a child. Even though, stroke#23 meets the criterion of being a control as defined in the protocol (see previous section of 2.1 Description of samples and datasets), we decided to remove both Stroke#61 and Stroke#23 from the dataset with the following justifications: (1) We considered our data cleaning process as a statistical outlier filter. Both Stroke#61 and Stroke#23 met the criteria as outliers; (2) The fact that we were able to discover a mistaken sample offered us confidence that the outlier filter was valid to a large extent. We believed it is reasonable to eliminate these two subjects from the dataset. All further analysis was based on the remaining training set of 47 stroke patients and 31 controls.

### 2.7. Evaluation of the LAD classification model

**Figure 3:**
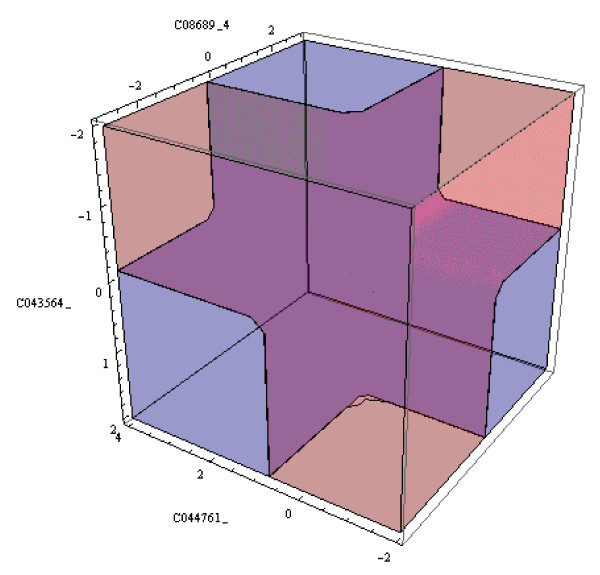
**A 3-D plot of the discriminant function on the training data.** The region colored pink (blue) represents the positively (negatively) classified region. The surface of the discriminant function is colored purple.

The LAD model can be used for diagnosing "new" observations, i.e. observations not included in the original dataset. If the observation satisfies *h *of the *p *positive patterns in the model, and *k *of the *n *negative patterns, then we define the "LAD discriminant function" as the expression *h/p - k/n*. The observation is classified based on the sign of the discriminant function. If *h/p - k/n *is equal to 0, the observation remains "unclassified". The LAD discriminant function corresponding to the training data and the LAD classification model in Table 3 is plotted in Figure 3. The region colored pink corresponds to the region where the discriminant function is evaluated to have positive sign, and hence an observation in this region will be classified as positive, while the blue region corresponds to the negative sign and hence it will classify observations as negative. Observations which lie on the surface of the discriminant function will be left unclassified.

According to the above definition, the sensitivity of a model is defined as the proportion of correctly classified stroke patients, while the specificity of a model is defined as the proportion of correctly classified controls. The accuracy of a model is defined as the average of its so-called "corrected sensitivity" and "corrected specificity", where the corrected sensitivity of a model is the usual sensitivity plus half of the proportion of unclassified stroke patients, and the corrected specificity of a model is the usual specificity plus half of the proportion of unclassified controls. Let *P*, *N *be the number of positive, respectively negative observations. Let *p*, *n *denote the number of correctly classified positive, respectively negative observations. Let *u*_*p*_, *u*_*n *_be the number of unclassified positive and negative observations respectively. The formula for sensitivity, specificity, corrected sensitivity, corrected specificity, and accuracy are presented below:

Sensitivity = p/P

Specificity = n/N

*Corrected sensitivity = p/P + 1/2(u*_*p*_/*P)*

*Corrected specificity = n/N + 1/2(u*_*n*_/*N)*

Accuracy = 1/2(Corrected sensitivity + Corrected specificity)

The accuracy, sensitivity, specificity and hazard ratio of the diagnostic LAD system applied to the stroke data are shown in the Table 4. We present the results of testing the model on the training set, then on the validation set, and finally the average results (with 95% confidence interval) over ten random 10-folding cross-validation experiments on the training set, where in each fold a model is built on a part of the training set and tested on the remainder.

**Table 4:** Performance of LAD model

	Performance	Training set	Validation set	Cross-validation
Logical Analysis of Data Model	Accuracy	82.6%	74.8%	79.8 ± 2.9%
	Sensitivity	89.4%	77.5%	85.4 ± 5.4%
	Specificity	74.2%	72.0%	70.6 ± 3.2%
	Hazard Ratio	8.1	3.2	3.0 ± 0.3

We have also computed the accuracy of other frequently used classification methods (C4.5 decision trees, Logistic regression, Support vector machines, and Multilayer perceptron) available in the Weka software package for the purpose of assessing the relative quality of our results. The control parameters of each of the other classification methods utilized were extensively calibrated to obtain the best performance (average accuracy in cross-validation experiments). These results are listed in Table 5. From Tables 4 and 5 we observe that the LAD model has significantly better performance on the independent validation set compared to the other classification models.

**Table 5:** Performance of other classification methods:

Method	Performance	Training	Validation	Cross validation
C4.5 Decision Trees	Accuracy	84.5%	69.3%	75.9 ± 2.9%
	Sensitivity	90.3%	76.0%	80.4 ± 4.8%
	Specificity	78.7%	62.5%	71.4 ± 3.5%

Logistic Regression	Accuracy	73.8%	64.8%	71.1 ± 1.8%
	Sensitivity	64.5%	52.0%	59.4 ± 2.7%
	Specificity	83.0%	77.5%	82.8 ± 3.3%

Support Vector Machines	Accuracy	81.8%	68.5%	77.6 ± 3.1%
	Sensitivity	74.2%	52.0%	87.50 ± 3.3%
	Specificity	89.4%	85.0%	67.80 ± 5.2%

Multilayer Perceptron	Accuracy	88.8%	68.5%	82.20 ± 2.4%
	Sensitivity	96.8%	72.0%	78.00 ± 3.5%
	Specificity	80.9%	65.0%	86.40 ± 3.7%

**Figure 4:**
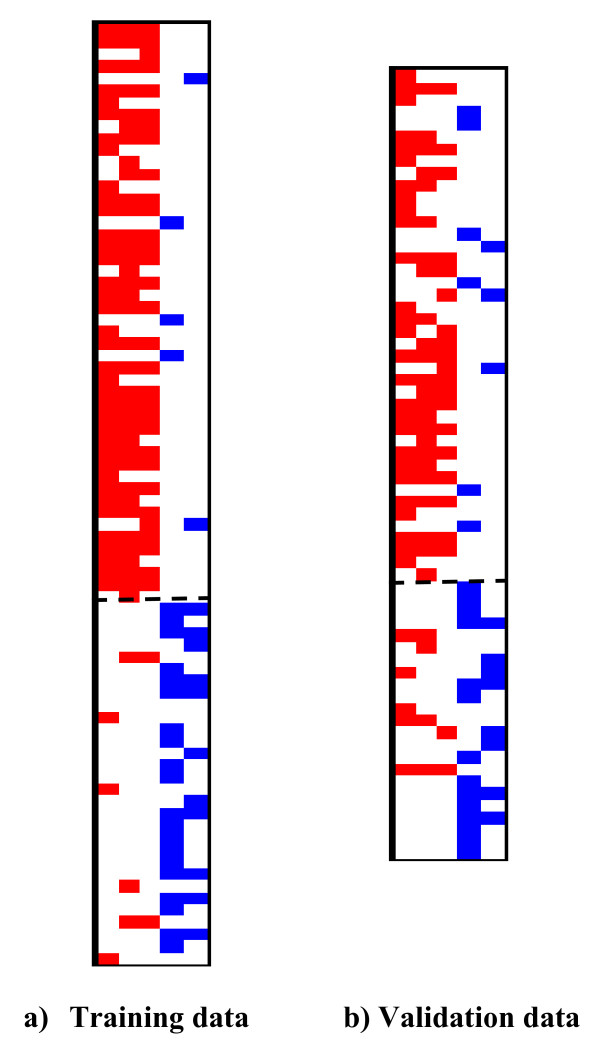
**Visualization of the pattern coverage on the training data (left) and test data (right).** Each row indicates an observation, and each column indicates a pattern. All observations above the dashed line are stroke patients, while those below the dashed line are controls. A cell corresponding to an observation *j *and positive (negative) pattern *p *is colored red (blue) if *j *is covered by pattern *p*.

To visualize the quality of the model on the observations in the training and test dataset we present Figures 4. These plots are visualizations of the coverage of observations by patterns in the model. The rows represent the observations with the positive observations above the horizontal dashed line, while the negative observations lie below the dashed line. The columns represent the pattern (first positive patterns followed by negative patterns). If an observation is covered by a positive pattern, then the corresponding pixel is coloured red, while if it is covered by a negative pattern it is coloured blue. From the two plots we can observe that the positive observations are covered by a majority of positive patterns, while the negative ones are covered by a majority of negative patterns. This is an indication of the high quality of the LAD model.

### 2.8. LAD-based severity score

To further evaluate our choice of a support-set, we attempted to build a model to predict the severity of stroke. When the patients were admitted to the hospital after a stroke, the stroke severity was evaluated by the physician using the National Institutes of Health Stroke Scale (NIHSS). NIHSS is used to measure the severity of neurological dysfunction at the time of a stroke [15]. A score greater than 25 indicates very severe neurological impairment; a score between 15 and 25 indicates severe impairment, while a score between 5 and 15 indicates mild to moderately severe impairment, and a score less than 5 indicates mild impairment.

We have identified a formula which can reasonably predict the severity score based on the knowledge of the 3 peaks (C08689_4, C043564_, and C044761_) on which the stroke diagnostic system was built. We have also experimented with several types of regression methods (Linear Regression, Multilayer Perceptron, Support Vector Regression, and LAD Regression). The results are summarized in Table 6, where the Pearson correlation column refers to the correlation between the predicted and the originally given severity scores, and the root mean square error (RMSE) is that of the predicted scores compared again with the originally given ones. Obviously, the ideal correlation is 1, and the ideal root mean square error is 0.

**Table 6:** Comparison of LAD Regression and other methods

	Training		Validation	
		
Method	Root Mean Square Error	Pearson Correlation	Root Mean Square Error	Pearson Correlation
Linear Regression	5.345	0.456	6.934	0.200
Multilayer Perceptron	5.941	0.394	6.696	0.209
Support Vector Regression	6.119	0.454	6.871	0.149
LAD Regression	3.158	0.851	5.900	0.450

It appears that both RMSE and the correlation show that the chosen support-set can provide (at least a partial) explanation of the level of the stroke severity score. LAD regression seems to provide the best results among the four methods. The stroke severity prediction of the LAD model for a patient *c *is given by the formula

*R(c) *= 8.61 - 4.63 * *Q1 *- 9.29 * *Q2 *- 4.69 * *Q3 *- 7.61 * *Q4 *- 6.09 * *Q5 *-6.11 * *Q6 *- 6.48 * *Q7 *+ 8.95 * *Q8 *+ 9.13 * *Q9 *+ 5.90 * *Q10 *+ 3.34 * *Q11*

where the values of *Q1*,...,*Q11 *are either 0 or 1 and are given by the formulas in Table 7.

**Table 7:** Formulas for *Q1,..., Q11 *in *R(c)*

*Q1 = 1 if I(C08689_4 > 0.274) = 1 AND I(C043564_ > -0.62) = 1*
*Q2 = 1 if I(-0.715 < C08689_4 ≤ -0.412) = 1 AND I(C044761_ ≤ -0.661) = 1*
*Q3 = 1 if I(C08689_4 > 0.066) = 1 AND (C043564_ > -0.62) = 1 AND I(C044761_ >-0.14) = 1*
*Q4 = 1 if I(-0.333 < C043564_ ≤ -0.221) = 1 AND I(C043564_ ≤ 0.710) = 1 AND I(C044761_ ≤ -0.14) = 1*
*Q5 = 1 if I(-0.221 < C08689_4 ≤ 0.066) = 1*
*Q6 = 1 if I(C08689_4 > 0.880) = 1 AND I(C043564_ ≤ -0.62) = 1*
*Q7 = 1 if I(C08689_4 ≤ -0.333) = 1 AND I(C044761_ > -0.661) = 1*
*Q8 = 1 if I(-1.21 < C08689_4 ≤ -0.636) = 1 AND I(C044761_ > -0.27) = 1*
*Q9 = 1 if I(C08689_4 ≤ -1.832) = 1 AND I(C043564_ ≤ -0.567) = 1*
*Q10 = 1 if I(-1.082 < C08689_4 ≤ 0.880) = 1 AND I(-0.748 < C044761_ ≤ -0.27) = 1*
*Q11 = 1 if I(-1.21 < C08689_4 ≤ -0.636) = 1 AND I(C043564_ ≤ -0.567) = 1*

For illustration, consider the case of stroke patient Stroke#80 having the following measurements: C08689_4 = -1.098, C043564_ = 1.547 and C044761_ = 3.112. For this patient *Q1 *= 0, *Q2 *= 0, *Q3 *= 0, *Q4 *= 0, *Q5 *= 0, *Q6 *= 0, *Q7 *= 1, *Q8 *= 1, *Q9 *= 0, *Q10 *= 0, and *Q11 *= 0. Therefore, *R(Stroke#80) *= 8.61 - 6.48 + 8.95 = 11.08. The original severity score of Stroke#80 was 12.

### 2.9. Classification and regression software

The LAD results described in this study were obtained with the use of "Ladoscope" [16], a publicly available implementation of LAD, written and maintained by Pierre Lemaire. Each of the steps involved in the construction of a LAD classifier (discretization, feature selection, pattern generation, model selection, and classification), as described in this study, are available in Ladoscope. The remaining classification methods (Support vector machines, C4.5 decision trees, Logistic regression and Multilayer perceptron) as well as the regression methods (Support vector regression, linear regression) utilized for comparison are available in the Weka package [17]. The LAD regression algorithm used for developing the LAD-based severity score formula was implemented by the authors and a public version of it is currently being prepared.

## Discussion

The main emphasis of this study has been to identify a small subset of variables that could allow the development of accurate and simple models for predicting stroke. The basic assumption being that a simple model would provide an easy and intelligible tool for medical experts to identify the stroke status of the patients by applying a small number of simple tests, such as drawing some blood, and performing the tests necessary to measure the levels of a few biomarkers.

On the value of predicting stroke or the intensity of the stroke using these models, it is conceivable that patients with acute stroke syndromes and cardiovascular instability (i.e. shock, arrhythmias) may have acute phase protein release including inflammatory mediators that may confound the protein expression pattern observed in the current study cohort.

We have described a detailed pre-processing algorithm for retaining important peaks. While the pre-processing procedure utilized was somewhat involved, it utilized a series of steps that in principle are not biased towards any of the classification methods applied to the problem: 1) removal of peaks with weak spectrum quality and peaks that were not reproducible across experiments, or did not have a significant individual predictive power; 2) removal of variables that did not show a good predictive power when utilized in pairs with other variables. The support-set of 3 peaks was chosen to improve the accuracy of classification in cross-validation experiments. In our early experiments, all classification methods (including LAD) overfit the training data when the support-set consisted of a large numbers of variables. While it is conceivable that a careful selection of variables would benefit other algorithms and deteriorate LAD performance, we did not experience this in our experiments. We notice here that the support-set selection procedure utilized was somewhat biased to the way in which the LAD and C4.5 classification algorithms work. While this choice may favor these two algorithms, we have observed a consistent increase in accuracy over all algorithms when utilizing the 3-peak support set. Moreover, we believe that a detailed evaluation of algorithms for feature selection is beyond the scope of the present study.

The strengths of the classification model proposed in this study are: a) small sized support-set, b) small number of patterns, c) interpretability and d) high accuracy on validation. The three peaks in the support-set identified, along with the patterns produced, can be seen as biomarkers, and their performance in predicting stroke qualifies them as research hypotheses for biologists and stroke specialists. This study demonstrates the potential of such a technique to help understand better the proteomic mechanisms behind stroke and its causes.

In addition to a classification model, we have been able to provide a predictive model to predict the intensity of a stroke, as defined by the NIH Stroke Scale. The scoring function built with LAD uses 11 rules constructed on the basis of the same 3 peptides in the support-set used for deriving the classification model of Table 2. This is an interesting fact in itself, since the original NIH Stroke Scale is constructed on the basis of clinical measurements and on subjective evaluation by medical doctors. The derivation of such a simple function that provides a good approximation to the standard NIHSS suggests the potential of this technique towards the construction of an automatic index that could provide valuable information regarding the severity of the stroke. Clearly, the use of more peaks could potentially improve the correlation and mean squared error on the training set, but this would most likely produce inferior results on validation, due to the small size of the training set. A rule of thumb for regression experiments is to have at least 8 to 10 times as many observation points as predictor variables. Since each rule can be regarded as an individual (combinatorial) predictor – taking values 0 or 1 at a given observation depending on whether the rule is satisfied or not on that observation – the set of 11 rules generated was just about enough to comply with this general requirement. Indeed, the predictive model was observed to provide a reasonably accurate prediction for the validation dataset, in spite of the limited training data.

In this study, LAD has been applied on a small sized dataset. In general, LAD can be scaled very well to large datasets. It has been successfully applied to studies with a large number of patients [11]. The combinatorial nature of LAD renders it sensitive to a large number of variables, just as other algorithms, such as decision trees. In such cases, feature selection has simplified the LAD computations and showed that a significantly small support-set is usually enough to construct LAD models of superior performance. A detailed description of different pattern generation algorithms along with the complexity is presented in [18-21].

## Conclusion

We successfully identified 3 biomarkers that can detect ischemic stroke with an accuracy of 75%. Table 3 shows that the performance of the classification model on the validation set and on cross-validation does not deteriorate significantly when compared to that on the training set. This is more evident when we compare with the decrease in accuracy observed in the LAD experiments with the experiments using other classification algorithms, shown in Table 4. This illustrates the robustness of our results.

As in the case of the LAD classification model, the regression results presented in Table 5 validates the function constructed on our support-set for approximating the severity scores of stroke patients. The correlation and root mean absolute error of the LAD regression are consistently superior to those of the other algorithms used. The decrease in correlation from training set to validation set is smaller than that observed in the results of the other algorithms.

## Authors' contributions

AR, TOB and VG were involved in analyzing the dataset, building the LAD classification models, and the LAD regression models. This analysis was supervised and approved by PLH.

HW, HY, JA, GH were involved in collecting the samples in the dataset, processing the samples for the SELDI analysis, SELDI MS acquisition, data processing with CiphergenExpress and in verifying the quality of the peaks (section 2.3). Their work was supervised and approved by KCL and AEB.

## Pre-publication history

The pre-publication history for this paper can be accessed here:



## References

[B1] Petricoin EF, Ardekani AM, Hitt BA, Levine PJ, Fusaro VA, Steinberg SM, Mills GB, Simone C, Fishman DA, Kohn EC, Liotta LA (2002). Use of proteomic patterns in serum to identify ovarian cancer. Lancet.

[B2] Alexe G, Alexe S, Axelrod DE, Bonates TO, Lozina I, Reiss M, Hammer PL (2004). Ovarian cancer detection by logical analysis of proteomic data. Proteomics.

[B3] Li J, Zhang Z, Rosenzweig J, Wang YY, Chan DW (2002). Proteomics and bioinformatics approaches for identification of serum biomarkers to detect breast cancer. Clin Chem.

[B4] Adam BL, Qu Y, Davis JW, Ward MD, Clements MA, Cazares LH, Semmes OJ, Schellhammer PF, Yasui Y, Feng Z, Wright GL (2002). Serum protein fingerprinting coupled with a pattern-matching algorithm distinguishes prostate cancer from benign prostate hyperplasia and healthy men. Cancer Res.

[B5] Allard L, Lescuyer P, Burgess J, Leung KY, Ward M, Walter N, Burkhard PR, Corthals G, Hochstrasser DF, Sanchez JC (2004). ApoC-I and ApoC-III as potential plasmatic markers to distinguish between ischemic and hemorrhagic stroke. Proteomics.

[B6] Lopez MF, Mikulskis A, Kuzdzal S, Bennett DA, Nelly J, Golenko E, DiCesare J, Denoyer E, Patton WF, Ediger R, Sapp L, Ziegert T, Lynch C, Kramer S, Whiteley GR, Wall MR, Mannion DP, Cioppa GD, Rakitan JS, Wolfe GM (2004). High-resolution serum proteomic profiling of Alzheimer disease samples reveals disease-specific, carrier-protein-bound mass signatures. Clin Chem.

[B7] Crama Y, Hammer PL, Ibaraki T (1998). Cause-effect relationships and partially defined Boolean functions. Ann Operations Res.

[B8] Boros E, Hammer PL, Ibaraki T, Kogan A (1997). A logical analysis of numerical data. Math Programming.

[B9] Boros E, Hammer PL, Ibaraki T, Kogan A, Mayoraz E, Muchnik I (2000). An Implementation of Logical Analysis of Data. IEEE Trans on Knowl and Data Eng.

[B10] Lauer MS, Alexe S, Snader CEP, Blackstone EH, Ishwaran H, Hammer PL (2002). Use of the logical analysis of data method for assessing long-term mortality risk after exercise electrocardiography. Circulation.

[B11] Alexe S, Blackstone EH, Hammer PL, Ishwaran H, Lauer MS, Snader CEP (2003). Coronary risk prediction by Logical Analysis of Data. Ann Operations Res.

[B12] Abramson SD, Alexe G, Hammer PL, Kohn J (2005). A computational approach to predicting cell growth on polymeric biomaterials. J Biomed Mater Res A.

[B13] Alexe G, Alexe S, Axelrod DE, Bonates TO, Lozina I, Reiss M, Hammer PL (2006). Breast cancer prognosis by combinatorial analysis of gene expression data. Breast Cancer Res.

[B14] Hammer PL, Bonates TO (2006). Logical Analysis of Data: From Combinatorial Optimization to Medical Applications. Ann Operations Res.

[B15] Baird A, Dambrosia J, Janket S, Eichbaum Q, Chaves C, Silver B, Barber P, Parsons M, Darby D, Davis S (2001). A three-item scale for the early prediction of stroke recovery. Lancet.

[B16] Lemaire P The ladoscope gang: Tools for the Logical Analysis of Data. http://www.kamick.org/lemaire/LAD/.

[B17] Ian HW, Frank E (2005). Data Mining: Practical machine learning tools and techniques.

[B18] Hammer PL, Kogan A, Simeone B, Szedmák S (2004). Pareto-optimal patterns in logical analysis of data. Discrete Appl Math.

[B19] Alexe S, Hammer PL (2006). Accelerated algorithm for pattern detection in logical analysis of data. Discrete Appl Math.

[B20] Alexe G, Hammer PL (2006). Spanned patterns for the logical analysis of data. Discrete Appl Math.

[B21] Bonates TO, Hammer PL, Kogan A (2008). Maximum patterns in datasets. Discrete Appl Math.

